# Exercise Tolerance and the Post Exercise Diastolic Filling Pattern in Patients With the Resting Impaired Relaxation

**DOI:** 10.4021/cr71w

**Published:** 2011-07-25

**Authors:** Steven J. Lavine, Thomas Walsh

**Affiliations:** a Wayne State University, Detroit, MI and University of Florida College of Medicine-Jacksonville, Jacksonville, FL, USA

**Keywords:** Impaired relaxation, Exercise, Diastolic filling, Transmitral filling patterns

## Abstract

**Background:**

In patients with normal LV systolic function, cardiac output increases with exercise mediated by increased stroke volume early in exercise and an increase in heart rate later in exercise. Despite normal LV systolic function, patients who display an impaired relaxation pattern may have a reduced exercise tolerance. We hypothesized that the resting impaired relaxation pattern that persists during exercise results in reduced LV filling volume and reduced exercise tolerance.

**Methods:**

We evaluated consecutive exercise echocardiograms performed at Harper Hospital from 1998-2000 for patients with sinus rhythm, normal resting wall motion and ejection fraction (> 55%), evidence of resting impaired relaxation, and a negative exercise echocardiogram. There were 49 patients fitting the above criteria who were compared with a group of age and sex matched patients (43 patients) with a normal rest and exercise echocardiogram with normal resting transmitral Doppler. Rest and post exercise echocardiography and Doppler parameters were obtained.

**Results:**

Patients in the impaired relaxation group demonstrated shorter exercise times as compared to the normal control group (8.8 ± 1.6 versus 9.7 ± 2.0 minutes, P < 0.001). In patients with normal resting transmitral diastolic filling, there was an increased the extent of atrial contribution to LV filling volume post exercise associated with shortening of isovolumic relaxation. Two patterns were seen in the impaired relaxation group post exercise. In 1 subgroup in which E/A ratio decreased post exercise, exercise duration was reduced (7.4 ± 1.3 minutes, P < 0.001) as compared to the subgroup with E/A increase (9.6 ± 1.2 minutes) post exercise which was similar to normal controls. Forward stepwise regression indicated that exercise time was primarily related to E/A change post exercise for all patient groups (r = 0.625, P = 0.0008). Specifically, this was true for patients with E/A reversal at rest (r = 0.584, P = 0.0028). However, for patients with normal diastolic filling at rest, the diastolic velocity integral was the major predictor (r = 0.695, P < 0.0084).

**Conclusion:**

We conclude that the transmitral Doppler pattern post exercise provides insight into the mechanism of reduced exercise tolerance in some patients with the resting impaired relaxation pattern. Preservation of this pattern post exercise is associated with reduced exercise tolerance.

## Introduction

In patients with normal LV systolic function, cardiac output increases with exercise mediated by an increase in stroke volume and heart rate. Early in the course of exercise, stroke volume increases and plateaus while progressively increasing heart rate becomes responsible for additional cardiac output increases in the normal left ventricle [1-2]. Many patients display an impaired relaxation pattern associated with hypertension, LV hypertrophy, diabetes, increasing age, or coronary disease [3-7] despite normal LV systolic dysfunction. Exercise tolerance in patients with an impaired mitral Doppler relaxation pattern at rest has been variably described either as normal or reduced [8-9]. The addition of pulsed mitral annular Doppler rapid filling velocity (e’) has not always been helpful in identifying exercise tolerance [10-11]. However, an elevated ratio of the resting rapid filling transmitral Doppler velocity/pulsed mitral annular Doppler rapid filling velocity (E/e’) may be associated with reduced exercise capacity [10-11]. Unfortunately, the groups studied include patients being evaluated for dyspnea and hypertensive patients who were variably symptomatic.

More recently, the use of exercise mitral Doppler patterns and the exercise ratio of E/e’ have been utilized to assess exercise capacity but only in patient groups with normal systolic and diastolic function, reduced systolic function, and in patients who develop ischemia. These studies have demonstrated that both exercise transmitral Doppler and the exercise E/e’ pattern predicted reduced exercise tolerance [11-13].

In patients with normal systolic function and impaired relaxation, atrial compensation has already been invoked at rest to maintain filling volume [1]. It is not clear whether exercise in these patients will result in increases in stroke volume or whether cardiac output solely increases from heart rate. Accordingly, we hypothesize that the resting impaired relaxation pattern that persists during exercise results in reduced LV filling volume and consequently reduced exercise tolerance. For patients with normalization of the diastolic filling pattern with exercise, exercise capacity may increase despite possibly being limited by mildly elevated LV filling pressures [13].

## Methods

### Patients

This study was approved by the Wayne State University Human Investigation Committee (IRB) as an exempt study. We evaluated consecutive exercise echocardiograms performed at Harper Hospital from 1998-2000 for patients with sinus rhythm, normal resting wall motion and ejection fraction (> 55%), evidence of resting impaired relaxation on diastolic filling (peak rapid filling velocity/peak atrial filling velocity < 1 and deceleration time > 240 msec), no significant valvular regurgitation greater than mild of any of the 4 valves, and a negative ECG response to exercise associated with an improvement in exercise ejection fraction without evidence of a wall motion abnormality. As a routine since 1993, transmitral Doppler was recorded during recovery from treadmill exercise. The exercise echocardiograms were reviewed to ensure that diastolic filling pattern could be evaluated during the recovery period (within 2 minutes post exercise) consisting of visibly separate peak rapid filling (E) and peak atrial filling (A) waves. We performed 861 exercise echocardiograms over this 2-year period. After applying the inclusion criteria, there were 49 patients who fit these criteria. The indication for the exercise echocardiogram was a chest pain syndrome in 45 and dyspnea in 4. A second group of age and sex matched patients (43 patients) with a normal rest and exercise echocardiogram and normal resting transmitral Doppler pattern were selected from the same days that the impaired relaxation group was studied. The indication for the exercise echocardiogram was chest pain in 39 and dyspnea in 2, and palpitations in 2.

### Rest and Exercise Echocardiography

Rest and exercise echocardiography were obtained with a HP 2500 echocardiograph (Hewlett Packard, Andover, MA) interfaced to an offline digital acquisition and display system for LV recordings (Microsonics, Bothell, WA) and stress ECG recording system (Marquette-GE, Milwaukee, WI). Standard parasternal, apical, and subcostal views were obtained along with pulsed wave, continuous wave, and color Doppler across each valve. Transmitral Doppler was obtained at rest with a 5 x 5 mm sample volume just beyond the tips of the mitral leaflets and recorded at 100 mm/s. Similarly, transaortic Doppler was recorded from the apical 5 chamber or 3 chamber view with 5 x 5 mm sample volume just beyond the aortic leaflets and recorded at 100 mm/s. All recordings were made on ½ inch VCR tape, and rest LV images in the parasternal long, mid ventricular short axis and in the apical 2 and 4 chamber views were digitally obtained.

Exercise was performed using a treadmill employing the Bruce protocol. All patients were exercised to their symptom limited maximum (impaired relaxation group: fatigue in 45 and chest pain in 4; normal resting transmitral Doppler group: fatigue in 39 and chest pain in 4). Immediately post exercise, each patient was rapidly imaged in the supine position within 45-60 seconds in the apical 4 and 2 chamber and parasternal long and short axis views. After obtaining the above images both on VCR tape and digitally, transmitral Doppler was obtained at a sweep speed of 100 mm/second as above on VCR tape at held end expiration within 2 minutes post exercise when distinct E and A waves of transmitral spectral Doppler had separated.

### Demographic Variables

The incidences of hypertension (blood pressure > 140/90 or on anti-hypertensive medications), diabetes (fasting blood sugar > 126 mg or on medications for glycemic control), and LV hypertrophy (LV mass index < 96 g/m^2^ in women and < 115 g/m^2^ in man) were determined for both the normal transmitral filling group and the impaired relaxation group.

### Exercise Testing Variables

Heart rate at rest, peak exercise, and at the time of transmitral Doppler recording was obtained. Resting and peak systolic and diastolic pressures were obtained. Double product at rest and peak exercise was obtained by multiplying heart rate by peak systolic pressure.

### Left Ventricular Volumes and Mass

LV volumes at end diastole and end systole at rest and with exercise were determined using biplane Simpson’s rule. LV mass was calculated from resting 2 dimensional echo data using the recommendations of the American Society Echocardiography [14] and indexed to body surface area.

### Doppler Variables

All calculations were from the average of 3 consecutive cycles. Color flow assessment of all 4 valves was performed. Any patient with valvular regurgitation greater than mild was excluded. Mild mitral regurgitation was defined as the ratio of the maximal color flow jet area/corresponding left atrial area < 20% in all apical views. Mild aortic regurgitation was defined as height of the aortic regurgitation jet in the LV outflow tract/LV outflow tract < 25% [15]. Mild tricuspid regurgitation was defined in a similar fashion to mild mitral regurgitation as a jet area of < 20% of the right atrial area in the apical 4 chamber view. Mild pulmonic regurgitation was assessed by the length of the jet in the RV outflow tract < 10 mm [15].

For both rest and post exercise, all Doppler indices were measured from the average of 3 consecutive cycles. From transmitral Doppler indices, peak rapid filling velocity (E) and peak atrial filling velocity (A) were measured. The rapid filling deceleration time was calculated as the time interval from the peak rapid filling velocity to the time mitral flow decelerated to the zero baseline. The tracing was extrapolated to the zero baseline if atrial filling commenced prior to mitral flow fully decelerating to zero. Diastolic filling, rapid filling, and atrial filling velocity integrals were determined. The length of the diastolic filling period was obtained as the interval from beginning to the end of transmitral spectral tracing. The atrial filling period and its integral were determined from the onset to the end of atrial filling. When rapid and atrial filling velocity spectra demonstrated any degree of merging, the onset of atrial filling was defined at the point of the end of the p wave on the ECG. The time from the R wave to the onset of the mitral time velocity spectrum was obtained. The time from the R wave to the end of rapid filling was obtained. If the rapid filling velocity had not decelerated to the baseline, then the point at which the velocity began to increase was use as the end of the rapid filling period. The rapid filling period and its integral were calculated from the onset of mitral inflow to the end of the rapid filling period. Isovolumic relaxation time was calculated as the time interval from the end of aortic velocity spectrum to the onset of the mitral velocity spectrum.

### Observer Variability

Intraobserver and interobserver variability was determined in 10 randomly selected patients for E, A, and the diastolic time velocity integral at rest and following exercise 3-7 weeks apart. For E, the difference for intraobserver and interobserver measurements were 2 ± 2 cm/s and 3 ± 2 cm/s at rest and 3 ± 3 cm/s and 3 ± 4 cm/s post exercise. For A, the difference for intraobserver and interobserver measurements were 2 ± 2 cm/s and 2 ± 3 cm/s at rest and 3 ± 3 cm/s and 3 ± 5 cm/s post exercise. For the diastolic time velocity integral, the difference for intraobserver and interobserver measurements were 0.09 ± 0.11 cm and 0.13 ± 0.11 cm at rest and 0.17 ± 0.19 cm and 0.20 ± 0.17 cm post exercise.

### Statistics

All continuous variables are expressed as mean ± standard deviation. Discrete variables are expressed as a percentage of the group. Comparisons between each group for discrete variable were performed with Chi square or Fisher exact test. Comparisons between groups or subgroups for continuous variables were performed with unpaired T tests. Comparisons between rest and exercise in a given group was performed with analysis of variance with repeated measures. Bonferonni’s correction was applied to multi-comparison T-test when the significance of the F value was < 0.05. Independent determinants of the exercise time and E/A change with exercise were performed with forward stepwise regression. All univariate variables of exercise time and E/A change with exercise with a P value < 0.10 were included.

## Results

Table 1 summarizes demographics and exercise testing variables for patients with E/A reversal (impaired relaxation) at rest and patients with normal diastolic filling. Patients with impaired relaxation exercised a shorter period of time, achieved a lower peak heart rate, and had an increased incidence of hypertension and LV hypertrophy. Diabetes was more frequent in patients with impaired relaxation. The effect of exercise on hemodynamic and transmitral and transaortic Doppler variables in patients with and without E/A at rest are summarized in Table 2. Peak exercise heart rate was higher in the group with normal diastolic filling at rest. Blood pressure response to exercise was similar in both groups. The impaired relaxation group demonstrated an increased peak atrial filling velocity, prolongation of atrial filling, increased atrial filling velocity integral, prolonged deceleration time, and a prolonged isovolumic relaxation time.

**Table 1:** General Characteristics of Patients With and Without E/A Reversal at Rest

General Characteristics	E/A Reversal at Rest (n = 49)		No E/A Reversal at Rest (n = 49)	P values
Age (yrs)	46 ± 11		42 ± 13	0.623
Male (%)	67%		77%	0.041
Resting Heart Rate	76 ± 11		73 ± 11	0.426
Peak Exercise Heart Rate (beats/min)	148 ± 26		164 ± 15	0.008
Peak Systolic Blood Pressure (mm Hg)	149 ± 18		156 ± 10	0.664
Exercise Time (min)	8.8 ± 1.6		9.7 ± 2.0	0.0007
Left Ventricular Hypertrophy (%)	34		0	0.006
Hypertension (%)	35		0	< 0.0001
Diabetes mellitus (%)	23		0	0.036
LV End Diastolic Volume (cc)	119 ± 33		112 ± 32	0.518
LV End Systolic Volume (cc)	46 ± 28		44 ± 25	0.712
Ejection Fraction (%)	62 ± 9		61 ± 10	0.862
E/A	0.78 ± 0.11		1.41 ± 0.24	< 0.0001
Deceleration Time (msec)	266 ± 32		196 ± 26	0.008

E/A: Peak rapid filling velocity/peak atrial filling velocity.

**Table 2:** Changes With Exercise in Patients With and Without E/A Reversal at Rest

Doppler variables	E/A Reversal at Rest		No E/A Reversal at Rest	
	Rest	Exercise	Rest	Exercise
HR (beats/min)	76 ± 11	148 ± 26^***^	73 ± 11	164 ± 15^***x^
SBP (mmHg)	128 ± 15	149 ± 18^**^	119 ± 9	156 ± 10^**^
DBP (mmHg)	81 ± 12	78 ± 12	70 ± 5^∧^	69 ± 4
E (cm/s)	66 ± 20	79 ± 22^**^	73 ± 13	96 ± 23^**x^
RVI (cm)	9.8 ± 4.3	9.2 ± 4.6	11.7 ± 3.3	13.5 ± 5.6^xx^
RFP (msec)	201 ± 50	158 ± 46^***^	231 ± 63	174 ± 48^***^
RFP/RR	0.25 ± 0.05	0.25 ± 0.05	0.27 ± 0.05	0.28 ± 0.06
A (cm/s)	85 ± 23	92 ± 21	54 ± 10^∧∧∧^	86 ± 23^***^
E/A (m/s)	0.78 ± 0.11	0.85 ± 0.13	1.41 ± 0.24^∧∧∧^	1.14 ± 0.19^**xxx^
AVI (cm)	9.5 ± 3.3	8.8 ± 2.9	4.5 ± 1.2^∧∧∧^	7.0 ± 1.8^**x^
DVI (cm)	19.7 ± 6.8	18.4 ± 6.8	17.5 ± 3.3	19.9 ± 3.5
AFP (msec)	152 ± 33	128 ± 25^***^	112 ± 31^∧∧^	106 ± 22^xx^
AFP/RR	0.19 ± 0.04	0.21 ± 0.03^*^	0.13 ± 0.03^∧∧^	0.16 ± 0.05^*^^x^
DFP (msec)	417 ± 109	319 ± 107^***^	463 ± 134	440 ± 212^xx^
DFP/RR	0.51 ± 0.07	0.50 ± 0.07	0.54 ± 0.11	0.69 ± 0.29^*^^xx^
DCT (msec)	266 ± 32	261 ± 38	196 ± 26^∧∧^	211 ± 32^xx^
IRT (msec)	87 ± 41	66 ± 46^*^	69 ± 19	28 ± 24^*^^xx^
IRT/RR	0.11 ± 0.06	0.11 ± 0.07	0.07 ± 0.02^∧^	0.05 ± 0.04^*^^x^
RR (msec)	810 ± 127	632 ± 135^***^	855 ± 158	620 ± 97^***^

HR: heart rate; SBP: systolic blood pressure; DBP: diastolic blood pressure; E: peak rapid filling velocity; RVI: rapid filling integral; RFP: rapid filling period; RR: cycle length; A: peak atrial filling velocity; AVI: annular velocity integral; DVI: diastolic velocity integral; AFP: atrial filling period; DFP: diastolic filling period; DCT: deceleration time; IRT: isovolumic relaxation time.

*P < 0.05; **P < 0.01; ***P < 0.001 versus Rest; ∧P < 0.05; ∧∧P < 0.01; ∧∧∧P < 0.001 E/A rest versus No E/A reversal rest; ^x^P < 0.05; ^xx^P < 0.01; ^xxx^P < 0.001 E/A reversal with exercise versus No E/A reversal with exercise.

**Figure 1:**
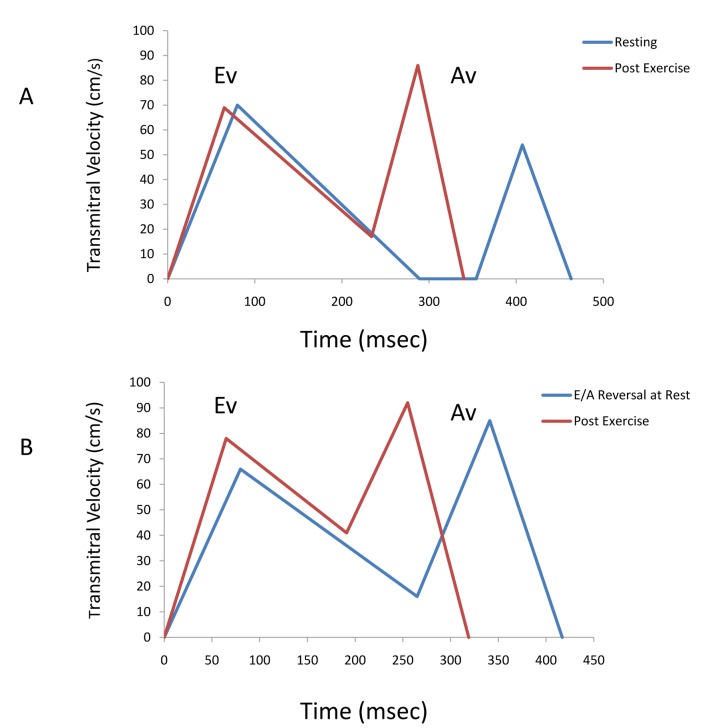
(A) A composite average transmitral flow in patients with normal diastolic filling is plotted against time both at rest and post exercise. Post exercise, there is an increased atrial filling velocity and atrial filling velocity. (B) A composite average transmitral flow in patients with E/A reversal at rest (impaired diastolic filling) is plotted against time both at rest and post exercise. Post exercise, there is an increased rapid filling velocity only and the the mean E/A remains < 1. Ev = Peak rapid filling velocity; Av = Peak atrial filling velocity.

Immediately post exercise (Table 2 and Fig. 1), the normal diastolic filling group demonstrated increases in the peak rapid filling and atrial filling velocities with a reduction in the E/A due to a greater increment in the peak atrial filling velocity. The diastolic filling period as a function of cycle length prolonged, and the isovolumic relaxation period as a function of cycle length shortened. Immediately post exercise, the impaired relaxation group increased peak rapid filling velocity, increased E/A compared to rest, and prolonged the atrial filling period. When comparing the impaired relaxation group vs the normal diastolic filling group post exercise, there was a lower peak rapid filling velocity, a smaller rapid filling velocity integral, a lower E/A, a larger atrial velocity integral, and a longer atrial filling period as a function of cycle length. There was also a longer deceleration time and isovolumic relaxation time as a function of cycle length despite a shorter diastolic filling period as function of cycle length. A graphic depiction of the transmitral filling pattern at rest and post exercise is shown in Fig. 1 for the 2 groups.

Table 3 summarizes the changes in diastolic filling from rest to post exercise in patients with normal and impaired diastolic filling at rest. Patients with normal diastolic filling demonstrated a greater increase in peak atrial filling velocity and integral, diastolic velocity integral, but associated with a reduction in E/A as compared to patients with impaired relaxation at rest.

**Table 3:** Transmitral Doppler Changes With Exercise in Patients With and Without E/A Reversal at Rest

Transmitral Doppler Changes	E/A reversal at Rest	No E/A Reversal at Rest	P Values
HR Change	+ 72 ± 21	+ 91 ± 16	0.009
RVI Change (cm)	- 0.7 ± 4.4	+ 1.8 ± 5.9	0.092
E Change (cm/s)	+ 12 ± 21	+ 23 ± 18	0.456
A Change (cm/s)	+ 8 ± 22	+ 32 ± 18	0.0006
EA Change	+ 0.08 ± 0.15	- 0.27 ± 0.25	< 0.0001
AVI Change (cm)	- 0.8 ± 3.0	+ 1.4 ± 2.4	0.009
DVI Change (cm)	- 1.4 ± 6.6	+ 2.4 ± 3.7	0.035
DFP/RR	- 0.1 ± 0.5	0.15 ± 0.16	0.006

Abbreviations See Table 2 for abbreviations.

**Figure 2:**
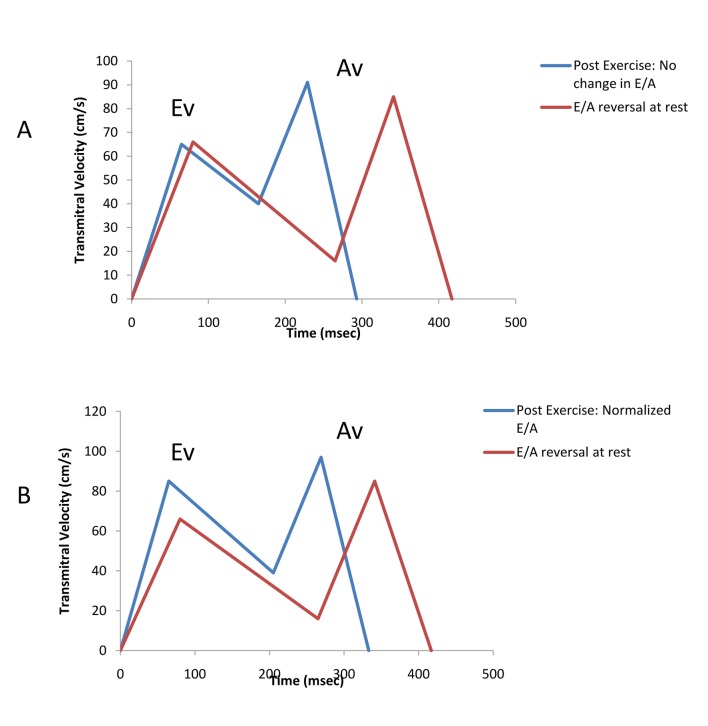
(A) A composite average transmitral flow in patients with E/A reversal at rest with no change in the E/A ratio post exercise. (B) A composite average transmitral flow in patients with E/A reversal at rest with an increased E/A post exercise due to a greater degree of augmentation in the peak rapid filing velocity. Ev = Peak rapid filling velocity; Av = Peak atrial filling velocity.

In patients with impaired relaxation filling pattern at rest, there were 2 separate and distinct responses in the diastolic filling pattern post exercise (Table 4). In 17 patients there was no change or a decrease in E/A post exercise (Fig. 2a) and in 32 patients, E/A increased post exercise (Fig. 2b). Exercise time was longer in the subgroup with an increase in E/A and a higher percentage of males were noted. The increase in E/A was primarily due to an increase in rapid filling as characterized by a greater change in the peak rapid filling velocity and in the rapid filling velocity integral than in the subgroup with no change or decrease in E/A.

**Table 4:** Effects of Exercise in Patients With E/A Reversal at Rest on Transmitral Doppler Parameters Who Developed Either an E/A Increase or Decrease With Exercise

Transmitral Doppler Parameters	E/A Increase with Exercise (n = 32)	E/A Decrease with Exercise (n = 17)	P value
Peak HR Change (beats/min)	72 ± 22	75 ± 21	0.886
% Male	50%	27%	0.024
Age (yrs)	56 ± 10	52 ± 13	0.715
Exercise Time (min)	9.6 ± 1.2	7.4 ± 1.3	0.0009
LVH (%)	33	38	0.716
HBP (%)	35	33	0.881
E Change (cm/s)	+ 19 ± 21	- 1 ± 16	0.009
RVI Change (cm)	+ 7.8 ± 3.7	- 3.4 ± 4.5	0.008
A change (cm/s)	+ 12 ± 25	+ 6 ± 20	0.533
EA Change	+ 0.16 ± 0.12	- 0.06 ± 0.08	< 0.0001
AVI Change (cm)	- 1.1 ± 3.1	- 0.2 ± 2.9	0.244
DVI Change (cm)	- 0.3 ± 6.6	- 3.4 ± 6.3	0.182
DFP Change (msec)	- 84 ± 77	- 124 ± 84	0.162
IRT Change (msec)	- 25 ± 51	- 3 ± 46	0.133

Abbreviations please see above tables.

Using forward stepwise multiple regression (Table 5), the independent determinants of E/A change post exercise demonstrated that exercise time and rapid filling velocity integral change were independent determinants of E/A change (r = 0.701, R^2^ = 0.491, P < 0.0001). Restricting the analysis to patients with impaired relaxation at rest demonstrated the same determinants (r = 0.727, R^2^ = 0.529, P < 0.0001). Similarly, the only independent determinant of exercise time for all groups (r = 0.625, P = 0.0008) and specifically in patients with impaired relaxation at rest was E/A change (r = 0.584, P = 0.0028). In patients with normal diastolic filling pattern at rest, exercise time was predicted by the diastolic velocity interval change (r = 0.695, P = 0.0084) which is an estimate of stroke volume.

**Table 5:** Forward Stepwise Regression

Determinants	Individual Correlation	Stepwise Correlation	Individual P value	Stepwise P value
Determinants of E/A Change in all Patients				
Exercise Time	0.598	0.598	0.0031	< 0.0001
RVI Change	0.469	0.701	0.0029	
Determinants of E/A Change in Patients with E/A Reversal at Rest				
Exercise Time	0.648	0.648	0.0002	< 0.0001
RVI Change	0.470	0.727	0.0189	
Determinants of Exercise Time in all Patients				
E/A Change	0.625	0.625	0.0008	0.0008
Determinants of Exercise Time in Patients with E/A Reversal at Rest				
E/A Change	0.584	0.584	0.0028	0.0028
Determinants of Exercise Time in Patients without E/A Reversal at Rest				
DVI Change	0.695	0.695	0.0084	0.0084

Abbreviations see previous tables.

## Discussion

In the normal patient, cardiac output increases with exercise mediated by an initial increase in stroke volume early in exercise with further increases in cardiac output resulting from increased heart rate [1-2]. However, many patients with normal pump function will display an impaired relaxation pattern on transmitral Doppler often related to hypertension, diabetes, coronary disease, age [3-7], and has been ascribed by clinicians as a cause of dyspnea. However, the E/e’ ratio and its correlation with brain natiuretic peptides has further cemented the relation of abnormal diastolic filling and elevated LV filling pressures as a cause of dyspnea and limitation of exercise [9-10]. More recently, the use of exercise mitral Doppler patterns and the exercise ratio of E/e’ have been utilized to assess exercise capacity but in patient groups with normal systolic and diastolic function, reduced systolic function, and in patients who develop ischemia. These studies have demonstrated that both the exercise mitral Doppler pattern and the E/e’ predict reduced exercise tolerance [9-13] based on increased E/e’ ratios. However, the role of increases in cardiac output with exercise has not been addressed in this patient group as a cause of “fatigue and dyspnea” as a limiting symptom.

In patients with normal systolic function and impaired relaxation, atrial compensation has already been invoked at rest to maintain filling volume. It is not clear whether exercise in these patients will result in increases in stroke volume or whether cardiac output solely increases from heart rate. Accordingly, we hypothesized that the resting impaired relaxation pattern that persists during exercise may result in reduced LV filling volume and contribute to reduced exercise tolerance.

In this study, we demonstrated that patients with normal resting transmitral diastolic filling patterns increased the extent of atrial contribution to LV filling volume post exercise associated with shortening of isovolumic relaxation, a finding previously demonstrated [16] suggesting the atrial systole is an important contributor to cardiac reserve during exercise. However, in patients with impaired diastolic filling, exercise time was reduced in this group as compared to the normal diastolic filling group. Subgroup analysis demonstrated that if an increased rapid filling contribution to filling volume occurred, a similar exercise tolerance was noted as compared to patients with normal diastolic filling at rest. Exercise tolerance was reduced in the subgroup whose diastolic filling pattern post exercise did not change and rapid filling contribution was similar to the resting pattern. Forward stepwise regression indicated that exercise time was primarily related to E/A change for all patients. Specifically, this was true for patients with E/A reversal at rest. However, for patients with normal diastolic filling at rest, the diastolic velocity integral was the best predictor. The strengths of these relations were moderate (r = 0.584-0.695). As we did not measure annular velocity with tissue Doppler (not available at the time of the study), we can not comment on the contribution of elevated LV filling pressures to exercise tolerance. For patients with normal resting filling, LV filling volume equivalent (or stroke volume) appeared to be a major determinant. Dyspnea and fatigue were a common cause of stopping exercise and may be related to either elevated LV filling pressures or limitations in cardiac output.

### Previous Literature

In patients with normal LV function, immediately post exercise, there is evidence of increased atrial contribution to filling volume suggesting that atrial systole is important for increasing stroke volume [1, 17, 18]. Impaired relaxation is seen in patients with coronary disease, hypertension, and LVH. These patients often demonstrate the transmitral pattern of impaired relaxation with prolonged isovolumic relaxation, E/A reversal, and prolonged deceleration time [3-7, 19]. Post exercise, the pattern noted may be variable. However, at rest, E/A reversal correlated with maximal O^2^ consumption in some patients with elevated LV filling pressures, decreased increment in cardiac index, and often LV systolic dysfunction [20]. In patients with hypertension and impaired relaxation, exercise capacity was reduced if isovolumic period did not shorten and if there was continued E/A reversal post exercise [9, 8]. As further diastolic dysfunction in hypertensive patients become evident with elevated LV filling pressures, workload correlated with natiuretic peptides a0"background: yellow"> E/E’ [11].

Nagueh demonstrated that a reduced peak annular filling velocity correlated with impairment of relaxation, and that the E/e’ ratio correlated with mean pulmonary capillary pressure [21]. In patients with heart failure, increasing E/e’ ratios correlated with increasing pulmonary capillary pressures and decreased O^2^ consumption [22-24]. Using a combination of transmitral filling and peak annular diastolic velocity at rest and post exercise in 179 patients referred for exercise echocardiography, exercise capacity was reduced in patients with impaired relaxation at rest and post exercise if there was an E/e’ > 11. Otherwise, a restrictive filling pattern at rest or post exercise resulted in reduced exercise capacity [12].

Our data indicates that E/A reversal at rest is associated with reduced exercise tolerance occurring in the subgroup with continued E/A reversal post exercise. This data is consistent with the LIFE sub-study [16]. Other studies indicate that E/A is inversely related to exercise tolerance, natiuretic peptides and the E/e’ ratios [8, 10, 13]. These patients often develop greater increases in LV filling pressures than likely in the LIFE sub-study. This is consistent with data from the diastolic stress testing in which post exercise increases in E/e’ correlate with reduced exercise capacity and increased natiuretic peptides [13]

### Limitations

This is a retrospective, single center study with limited numbers in each group. There was referral bias in our institution for exercise echocardiography as patients with lower coronary risk were more often studied using exercise echocardiography. Patients with a history of coronary disease, previous revascularization, or at high risk based on symptoms and risk profile more often-received nuclear perfusion studies. The inclusion and exclusion criteria for this study identified a cohort of patients in which the effect of impaired relaxation on exercise tolerance would have less confounding influences of a high cardiovascular risk profile. Despite the above limitations, our study demonstrates that patients with impaired relaxation by transmitral diastolic filling have reduced exercise tolerance and, specifically, when the transmitral Doppler filling profile post exercise continues to demonstrate E/A reversal. Finally, we did not record the transmitral filling profile during early exercise when the transmitral filling pattern was not fused. Different results may be possible.

### Clinical Implications

Recently, the use of E/e’ at rest and post exercise has been employed to determine whether dyspnea with exercise is related to elevated LV filling pressures [10-11, 13] The E/e’ ratio appears to predict elevated LV filling pressures at least at rest in patients with both reduced and preserved LV ejection fraction. The data post exercise appears to support the further use of this parameter as to a guide as to whether dyspnea as a limiting factor is due to elevation of LV filling pressures [10-11]. Ischemia with exercise can also manifest itself as dyspnea. This study specifically excluded these patients. Exercise tolerance may also be reduced due to fatigue due to a lesser increment in cardiac output with exercise. Fatigue may be difficult for patients to discern from dyspnea. Most patients in this study ceased exercise due to fatigue. We are unable to differentiate specifically whether LV filling pressures may have increased in these patients. However, the observation that atrial contribution to LV filling may be invoked in normals in this study suggest a limitation in the ability to increase stroke volume in patients with impaired relaxation at rest resulting in a heart rate increases to augment cardiac output. Not surprising, there are 2 distinct responses with preservation of the impaired relaxation pattern with reduced exercise tolerance as compared to the subgroup who utilized more rapid filling.

### Conclusion

We conclude that the transmitral Doppler pattern at rest and post exercise provides insight into the importance of the impaired relaxation pattern. Preservation of this pattern post exercise is associated with reduced exercise tolerance in a patient group with E/A reversal on transmitral Doppler referred for chest or dyspnea that continue to manifest this pattern post exercise. Further evaluation of this observation is indicated in groups with evidence of impaired relaxation: hypertension, valve disease, and diabetes.
